# Mutation patterns of integrase gene affect antiretroviral resistance in various non-B subtypes of human immunodeficiency virus Type-1 and their implications for patients’ therapy

**DOI:** 10.37796/2211-8039.1422

**Published:** 2023-12-01

**Authors:** Fauzia A. Djojosugito, Arfianti Arfianti, Rudi Wisaksana, Agnes R. Indrati

**Affiliations:** a Doctoral Study Program, Faculty of Medicine, Universitas Padjadjaran, Bandung, 40161, Indonesia; b Microbiology Department, Faculty of Medicine, Universitas Riau, Pekanbaru, 28133, Indonesia; c Medical Biology Department, Faculty of Medicine, Universitas Riau, Pekanbaru, 28133, Indonesia; d Research Centre for Care and Control of Infectious Disease, Universitas Padjadjaran, Bandung, 40161, Indonesia; e Internal Medicine Department, Hasan Sadikin Hospital, Bandung, 40161, Indonesia; f Clinical Pathology Department, Faculty of Medicine, Universitas Padjadjaran, Bandung, 40161, Indonesia

**Keywords:** HIV-1, Integrase, Resistance, Subtype non-B

## Abstract

Although primary integrase strand transfer inhibitor resistance mutations are currently uncommon, the increasing use of integrase strand transfer inhibitor as a key component of the first, second and third-line antiretroviral regimens suggests that the prevalence of integrase drug resistance mutations will likely increase. The rise of several polymorphic mutations and natural polymorphisms also affects the level of susceptibility of human immunodeficiency virus (HIV) type-1 to integrase strand transfer inhibitor. The considerable variability among the various subtypes of human immunodeficiency virus type-1 may contribute to differences in integrase mutations associated with integrase strand transfer inhibitors. Notably, non-B subtypes of HIV type-1 (HIV-1) are the predominant cause of human immunodeficiency virus infection worldwide. The presence of diverse integrase drug resistance mutations can have significant implications on the administration of integrase strand transfer inhibitor-based antiretroviral therapy to patients with human immunodeficiency virus infection.

## 1. Background

Drug resistance is dynamic and multifactorial, depending on the viral fitness of the variant, the degree of resistance conferred by specific mutations, and frequency of such mutations in the viral population [1]. Using a combination of antiretroviral (ARV) drugs provides good results in HIV/acquired immunodeficiency syndrome (AIDS) management, which causes viral suppression and prevention of transmission to enhance the quality of life of HIV patients [1–3]. In addition to nucleoside reverse transcriptase inhibitor (NRTI), integrase strand transfer inhibitor (INSTI) has been included as part of combination therapy for HIV patients, and may replace a non NRTI (NNRTI) [4]. The INSTI is active on viruses resistant to other ARV classes [5,6]. In addition, INSTI-based regimens are needed for treatment-naive HIV patients due to their high tolerance, favorable drug interactions and dosing, and a high genetic barrier to resistance [7].

Approximately 90% of the 35 million people infected with HIV-1 have non-B subtypes and recombinant forms of the virus [8]. The majority of HIV infections are caused by non-B subtypes, hence further data are needed to elucidate the natural polymorphisms of integrase associated with INSTI resistance in various HIV-variants [9,10]. It can affect genetic barriers and/or the INSTI resistance pathway, which could facilitate or delay a variant from becoming fully resistant to INSTI [10]. Therefore, natural polymorphisms of the integrase gene that occur in various HIV-1 subtypes can affect the development of INSTI resistance to varying degrees [5,11].

Due to the high variability among subtypes, specific HIV-1 subtypes can have different genetic drug resistance barriers [12]. In several studies, it has been stated that the virological outcome of therapy can be related to subtype-specific differences, therefore it is crucial to analyze the diversity of HIV subtypes contributing to drug resistance [12].

Increasing use of INSTI as a first-line regimen for HIV patients has led to the development of different genetic drug resistance barriers against INSTI drug resistance mutations. In various studies of newly diagnosed HIV patients in Europe, Canada, the United States, and China, major (primary) mutations were found in several strains of HIV-1 and polymorphic accessory mutations in the integrase gene [13]. Various reports have highlighted the presence of mutations in integrase and other genes that can cause resistance in all INSTI classes (pan integrase resistance) and mutations in other ARV classes that can impact the effectiveness of INSTI-based therapy [7,14]. In addition, the existence of resistance mutations has prompted careful consideration regarding the clinical use of INSTI, especially dolutegravir (DTG), whether in combination or as monotherapy [15].

This review aims to discuss the current understanding of the differences between major mutations and INSTI drug resistance mutation-associated polymorphisms among the various non- B subtypes of HIV-1 and the use of INSTI for ARV therapy in HIV-1 patients associated with the potential emergence of INSTI drug resistance.

## 2. Integrase enzyme and inhibitor

**Fig. 1:**
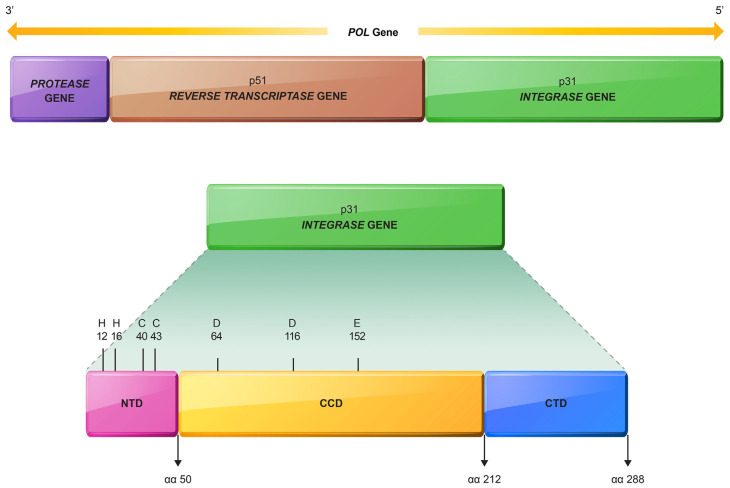
Pol and integrase genes. NTD = N-terminal zinc domain; CCD = Catalytic core domain; CTD = C- terminal domain DNA binding; aa = amino acid; H = histidine; C = cysteine.

Integrase (IN) is one of the HIV enzymes, encoded by integrase gene (at 5′ end of pol gene), which catalyzes the process of integrating HIV proviral DNA into host genome [16–19]. Integrase is a 288 amino acid (aa) protein consisting of 3 functional domains, namely the N-terminal zinc (NTD) highly conserved domain (aa 1–50), catalytic core domain (CCD) (aa 51–212) containing amino acids D (64) D (116) E (152) or DDE motif and plays an essential role in the activity of IN, and C-domain terminal DNA (CTD) binding (aa 213–288) which is involved in viral and cellular DNA binding, oligomerization proteins and interactions with reverse transcriptase (RT) (Fig. 1) [11,20–22]. Integration is a complex process of forming a permanent bond between viral genome and host genome consisting of several stages. The NTD binds to viral DNA sequences and plays a role in 3′ processing and strand transfer. The CCD, which contains the DDE motif, binds to the divalent magnesium cofactor. CTD interacts with RT and binds to DNA during integration [22,23].

The integrase inhibitor (INI) currently used is INSTI, which is the most recent ARV drug class to be approved and included in the World Health Organization (WHO) guidelines for HIV/AIDS treatment together with NRTI or as a salvage regimen for HIV patients who are resistant to the two main target enzymes of ARV drugs (RT and protease enzymes) [6,12,19,21,24,25]. INSTI is so named because it targets the integrase enzyme at the strand transfer stage by binding to two essential viral elements (integrase and viral DNA) [16]. INSTI is the latest drug to be developed with high efficacy, safety, and tolerability. The first generation drugs of INSTI, namely, raltegravir (RAL) and elvitegravir (EVG), are recommended for the initiation of ARV therapy [26]. RAL is classified under INSTI with a high need for daily pill consumption and low genetic barrier, which puts patients at risk for developing resistance mutations. On the other hand, EVG has more drug interactions than other INSTI drugs, which can make it challenging to use in certain patient populations [24,25]. Based on *in vitro* studies, cross-resistance is seen to occur often between RAL and EVG [5].

Therefore, after 2017, INSTI drugs for naive-treatment HIV patients were replaced by the second INSTI generation (DTG), and bictegravir (BIC) as first-line therapy regimens because they have a high rate of viral suppression, minimum toxicity, low drug interactions, high genetic barriers and are recommended as salvage regimens [25–27]. In some low and middle-income countries, a transition is taking place towards using combination ARV therapy as the first-line treatment. Accordingly, despite being second-line therapy, DTG is being used as the first-line regimen along with 2 NRTIs [11].

Other new INSTI in development is cabotegravir (CAB) that is still undergoing clinical trials [24,28,29]. The CAB drugs are formulated as long-acting injectable preparations, which are administered every month or every three months and as oral tablet preparations for daily use [12].

## 3. Non-B subtype of HIV-1

Due to the lack of proofreading mechanism of viral RT enzyme, fast viral replication, recombination in host cells, HIV has become a virus with high variability and mutation rate, resulting in the development of various subtypes of HIV [2,9,30]. Genetic variation within subtypes reached 8%–17%, even up to 30%, while variations between subtypes range from 17% to 35% [31]. Group M has nine subtypes/clades (A–D, F–H, J, and K) and is also known to have circulating recombinant forms (CRF), which currently have 98 CRFs and unique recombinant forms (URF) [32].

Although studies on HIV drugs and resistance mutations are dominated by the B subtype, it only accounts for 11%–12% of HIV-1 infections worldwide and non-B subtype dominates the global HIV epidemic [11,23,33,34]. Non-B subtype is a challenge for ARV therapy because of the presence of different groups and mutations. Their polymorphisms can cause changes in the structure of drug target’s viral proteins, thereby reducing the efficacy of ARV drugs and drug resistance development [9]. Some research indicates that polymorphisms occurring in non-B subtypes before therapy mean the development of subtype-specific secondary resistance [35].

These various subtypes of HIV-1 are distributed worldwide, and some subtypes become predominant in a given country region. Subtype B is commonly found in developed countries, Africa, and several countries in Southeast Asia, Russia and Middle East.^5^ On the Asian continent, there was an epidemic due to non-B and CRF subtypes [36]. Although HIV-1 molecular epidemic is a dynamic process, commonly the subtypes found worldwide are C (49.9%), A (12.3%), B (10.2%), G (6.3%), CRF01_AE (4.7%) and CRF02_AG (4.8%) [33].

## 4. Resistance mutations to INSTI

In the HIV-1 B subtype, there are 40 aa substitutions associated with INSTI resistance, most of which are located at integrase positions 66, 92, 143, 147, and 155 [5]. Resistance mutations to INSTI are almost always found at integrase active sites close to aa residues that interact with the magnesium cofactor, hence these mutations cause detrimental effects on the enzymatic function of integrase and the virus’s replicative capacity [37].

### 4.1. RAL-resistant integrase mutation

Integrase mutations Q148 H/R/K, N155H, and Y143 C/H/R are primary mutations and affect RAL resistance, especially if there are also secondary mutations [1,5,6,38–40]. The Q148 H/R/K and N155 mutations can cause cross-resistance against EVG, while the Y143 mutation was specific for RAL [1,5,6,40]. Failure of RAL therapy (*in vivo*) was associated with the genetic pathway, N155H mutation associated with secondary mutations L74M, E92Q, T97A, V151I, and G163R, and *in vitro* associated with secondary mutations G140 S/A and E138K [38,41].

In RAL therapy, selective pressure can cause non polymorphic accessory mutations during therapy. Natural polymorphisms in integrase genes such as V151I and I72V are linked to a slight decrease in susceptibility to INSTI [40]. Based on *in vivo* studies, the F121Y mutation, which has been shown *in vitro* to reduce susceptibility to RAL, has weak viral fitness, hence it was replaced by the 143R mutation. The results of this study also concluded that the F121Y mutation could mediate the development of the Y143R mutation [40,42].

### 4.2. EVG-resistant integrase mutation

Mutations T66I, E92QG, Q146P, and S147G are specific to EVG [1]. Mutations Q148 H/R/K, G140A, and N155H, which are known to cause resistance to RAL, also cause cross-resistance to EVG [1,6,40].

As observed *in vitro* studies, resistance to EVG is associated with primary mutations T66I and E92K that appear along with several secondary mutations such as H51Y, F121Y, S147G, S153Y, E157Q, and R263K. Whereas *in vivo*, there are several mutations associated with EVG resistance: E92Q with N155H and N155H with E138K, S147G, Q148R, G140 S/C, and Q148 R/H [41].

### 4.3. DTG-resistant integrase mutation

The INSTI DTG drugs are recommended by the WHO to be included as a component of second-third- line therapy regimens and also as one of the first-line regimens in many countries (either with or without a high prevalence of pretreatment drug resistance) [43]. Integrase enzymes can regulate reverse transcriptase activity at an early stage by increasing the reverse transcriptase action. In the integrase enzyme containing the R263K mutation, although it can affect the DNA-integrase interaction, in cell culture it does not change the reverse transcriptase enzyme action compared to wild-type integrase enzyme [6,44]. The INSTI-resistant mutations that are most often found in patients with virological failure who received DTG regimen are R263K, G118R, and Q148 H/R [5]. The R263K and G118R were mainly found in patients who were previously INSTI-naive. The R263K mutation reduces DTG susceptibility by 2-fold (low-level mutation), and G118R reduces DTG susceptibility by more than 5-fold. Clinically, resistance to DTG is often associated with the presence of specific mutations in integrase, such as the Q148 mutation, which reduces susceptibility to DTG as much as 10–20 fold when combined with two additional mutations at positions G140 and or E138 [28,45]. The DTG regimen in the form of a combination of three drugs is very effective as salvage therapy in naive INSTI patients [5–7,43].

The G118R mutation alone can cause high resistance to DTG and can cause pan integrase resistance [7]. Sequencing results show that the majority of the G118R mutations have a codon signature motif that undergo a point mutation, namely natural polymorphism of AGA at codon 118, requiring only one mutation to change AGA (G) to AGA (R) [1,7]. In addition, the presence of a major M184I/V NRTI mutation is thought to facilitate the emergence of the G118R mutation [7].

## 5. Integrase mutations and genetic barriers to drug resistance integrase in various non-B HIV-1 subtypes

Genetic codon diversity among various subtypes can lead to different genetic barriers to drug-resistance mutations. Various studies have shown that substitution of polymorphic or natural polymorphism (NOP), especially at the active site of integrase, causes natural integrase diversity among various variants of the HIV-1 subtype. This can significantly affect the genetic drug resistance barrier by influencing specific resistance mutations, native protein activity, viral replication capacity, and the function of antiretroviral drugs in inhibiting integrase enzymes. All of these impact INSTI therapy [11,12].

Natural polymorphisms in the HIV-1 integrase show high diversity of CTD integrases. Amino acid residues associated with catalytic activity (DDE motif) and major mutations in RAL and EGV (Y143, Q148, and N155) have been shown to be highly conserved among various subtypes. However, polymorphic variations were often found in secondary mutations such as V72, L74, T125, and M154 [22]. In a study in Russia, only a few major integrase mutations have been found, but polymorphic mutations were also observed which were significantly different between the A6 and A1 subtypes. These polymorphic mutations are K14R, R20K, L74I, S119P, T124S, T12A, V126F, G134N, T218I, L234I, S255N, and S283G [12].

In a study conducted in Cambodia, Thailand, and Vietnam in treatment-naive patients, it was found that the majority of the CRF01_AE subtype was the dominant subtype in Southeast Asia. A natural polymorphism of 41.3% of the total amino acid integrase residues was found on comparing subtype B sequences as reference sequences. The primary mutation associated with INSTI resistance was the E157Q mutation and was found in 1.1% of patients [21].

The Q148H and G140S mutations that can alter sensitivity to RAL and EVG and cause cross-resistance to DTG and R263K mutations are less common in non-B subtypes [46]. In contrast, G118R mutations that affect DTG resistance are more common in non-B subtypes [11]. The genetic barrier is an essential factor in the development of drug resistance and is determined by the cumulative number of DRM required by HIV to avoid drug selective pressure [34]. Various subtypes that differ at the nucleotide level can influence the genetic barrier against ARV [34]. In a study of treatment-naive INSTI patients in Ethiopia, genetic-barrier analysis found that the distribution of codons at the amino acid position associated with DRM in HIV subtype C had the same genetic barrier to DTG resistance as subtype B, except at codon position 140. Subtype C has a higher genetic barrier in the G140C and G140S mutations. The G140S mutation is a secondary mutation that can improve viral fitness caused by the Q148 mutation, resulting in a decrease in sensitivity to RAL and EVG by 130-fold and DTG by 3–8 fold. This means that the DTG Q148 H/K/R resistance mutation is rare in subtype C because the G140S mutation as a secondary mutation can repair the catalytic defects caused by the Q148H mutation so that G140S can improve viral fitness. The same thing happened to G140S or G140C mutations in the CRF02_AG and CRF01_AE subtypes [11,34]. Genetic-barrier G140S is higher in CRF01_AE, which means that CRF01_AE is less likely to develop resistance to RAL, EVG, and DTG in the presence of Q148S mutation than subtype B [34]. In contrast, studies on subtype C, predominantly found in Botswana, have shown that Q148R is one of the most commonly found INSTI DRM mutations besides E138K, S147G and N155H. This is because the highly treatment-experienced (average 11.5 years) subjects had been on DTG-based or RAL-based regimens and some had experienced VF due to the accumulation of DRM [46,47].

Research on genetic barriers by comparing sequences of subtype CRF01_AE with B and amino acid substitution of primary/major mutations resistant to RAL and EVG showed highly conserved between subtypes CRF01_AE and B. This means that those two subtypes have the same genetic barrier for RAL and EVG. In contrast, several secondary mutations to RAL, EVG, and DTG, namely V72I, L101I, A124T, T125K, and G140 C/S, showed high genetic barriers in HIV-1 CRF01_AE. In addition, this study found a variant containing the V201I mutation, which has a lower genetic barrier in CRF01_AE than subtype B [34].

A study of GenBank data until January 2015 on amino acid substitution analysis of 6706 sequences from INI-naive individuals of various HIV-1 subtypes, revealed either primary, secondary, or rare mutations. It was stated that INI primary (major) mutations were rarely found in these INSTI-naive patients and present at low prevalence (1–5 per variant) in subtypes B, C, D, CRF01_AE, and CRF14_BG. A primary mutation has not been found to be rare, but secondary mutations occur at various frequencies for each subtype. Minor/secondary mutations L74M, T97A, and G163RK, were found in more than 10% of INSTI-naïve patients. There are L74M in CRF43_02G (33.3%), T97A in group P (50%), J (33.3%), CRF18_cpx (20%), and F2 (11.5%). On the other hand, G163RK was found in large numbers in recombinant BF (CRF44_BF (100%), CRF46_BF (66.7%), CRF17_BF (28.6%), CRF12_BF (16.7%), and CRF29_BF (12.5%)) [10].

In addition, there are several minor INI mutations not found in all HIV-1 naive INSTI variants (V151LA, S230R), and INI mutations that are rare and associated with an insignificant effect on the integrase catalysis process (G118R, F121Y, P145S, and Q146P). There are several mutations, including natural polymorphisms found in more than 90% of non-B HIV-1 variant sequences (V72I, L101I, T124A, and E157Q) [10].

An *in vitro* study was conducted to determine the sensitivity of DTG to integrase enzyme containing R263K mutation in subtypes B and non-B (subtypes C, and A/G). It was found that these subtypes confer low resistance to DTG. The R263K mutation was the one most commonly found in the cultures of these various subtypes. In one of the B subtypes, there was a partial substitution of R263K with S153Y, raising speculations about the relationship between the substitution and resistance and viral fitness [6]. However, it requires further research. In addition, the G118R major mutation was found to occur more frequently in the non-B (C and A/G) subtypes than in the B subtype. The presence of the E138K secondary mutation could improve G188R viral fitness, and together with Q148K, it could also reduce the efficiency of DTG [1,6]. Various secondary mutations that appeared due to selective pressure *in vitro* were found (M501 and D288E), which are natural polymorphisms often found in clinical isolates. V151I polymorphic mutation is often found but does not cause a significant effect on the susceptibility of RAL and EVG [6].

In a study in Russia on ARV-treatment-naive and INSTI-treatment-naive patients, it was found that although mutations in INSTI-resistant resistance were low, the mutation frequency in INSTI-treatment- naive patients was higher than that in ARV-treatment-naïve patients (2.7% and 1.3%). The L74I mutation is a specific characteristic for the A6 subtype because, in a comparative analysis, position codon 74 differs significantly between A6 and other subtypes. However, the L74M and L74I mutations show the lowest genetic barrier in the A6 subtype. In addition, subtype B differs from subtype A at positions 140 and 151, and subtype B, which contains G140C and V151L mutations, has a lower genetic barrier that affects resistance to INSTI [12]. The L74I mutation is a polymorphic mutation that is found in a large proportion (21%) in subtype A sequences. Although the existence of an L74I mutation on INSTI susceptibility is still a matter of speculation, the L74I mutation is located near the integrase active site, so *in vitro* and clinical studies are needed to determine whether this polymorphic mutation has an impact on clinical and virological outcomes [12]. However, a study on treatment naive patients in Uganda found associations between certain HLA-genotype and L741I in subtype A1 infection [48].

The E92Q, Q148 K/R/H, N155H, and E157Q mutations, which are integrase mutations associated with resistance to RAL and EVG, are 77% conserved (highly conserved) between subtypes B and CRF02_AG and show the same barrier resistance between these two subtypes. Nevertheless, for specific mutations such as G140S, G140C, and V151I, CRF02_AG has higher scores than subtype B, which means that the presence of these mutations in CRF02_AG has a higher genetic barrier than in subtype B [5,41]. In addition, in biochemical studies, it was found that the presence of only E92Q mutation in HIV-1 virus subtype C did not lead to a significant difference compared to subtype B. If it contained only the N155H mutation, then the C subtype would be more susceptible (especially to EGV) than subtype B. However, if the E92Q mutation coexisted with N155H, then subtype C containing this double mutation will be 10-fold more susceptible to RAL and EVG than subtype B [5,22].

Based on the clinical trials phases 1 and 2 by comparing the clinical activity of RAL in subtype B and non-B subtype infections in the treatment-naive and experienced populations, RAL was active against all subtypes of HIV-1. In addition, in *in vitro* biochemical experiments, it was found that integrase in CRF02_AG and subtype C had little or no effect on susceptibility to INSTI [23].

An *in vitro* study was conducted to test the susceptibility of integrase enzyme in the HIV-1 strain subtype A and CRF01_AE to RAL by comparing it with integrase in subtype B [23]. This study also tested one of the major mutations in integrase, N155H mutation. The result revealed that integrase enzymes in subtypes A and CRF01_AE were susceptible to RAL, while the integrase enzyme in subtype B was susceptible to RAL. The R263K mutation reduces viral fitness and integration without affecting the action of the reverse transcriptase enzyme [23].

In a study conducted in Beijing, China, on HIV-1 naive-treatment patients, no major integrase mutations, but only 12 accessory polymorphic mutations were found. Among transmitted drug resistance (TDR) mutations, three were known to have INSTI low-level resistance, namely mutations E138A and G163R against RAL and EVG, and mutations S230R against RAL, EVG, DTG, and BIC. The majority of patients had integrase mutations with subtypes CRF01_AE, followed by CRF07_BC, CRF02_AG, and subtypes B and C [13].

## 6. INSTI drug resistance mutations and their implications in antiretroviral therapy

According to 2021 WHO guidelines, DTG in combination with other ARVs (NRTI) is recommended as a first-line INSTI regime [4] The RAL-based regimens are recommended for HIV-positive neonates, and low-dose EVG in combination with NRTIs is used as an alternative first-line regimen for HIV-infected adults and adolescents. The DTG plus NRTIs are recommended for adults, adolescents, and children with HIV [4] DTG should be used with caution in pregnant women due to the risk of neural tube defects in unborn babies, and their use in this group should be closely monitored [4,49]. In addition, results of several clinical trials and cohort studies indicate that patients receiving DTG therapy tend to gain weight compared to patients treated with evafirenz. This raises concerns about the side effects of DTG leading to obesity in the future [50].

There are concerns in administering DTG as monotherapy based on several *in vitro* research data, which states that there are differences in the barrier resistance pattern of DTG resistance compared to other INSTI (RAL and EVG). In DTG, high dissociation coefficient and low occurrence of mutants containing major mutations of INSTI compared to other INSTI indicate the efficacy of DTG as monotherapy [27]. In another study involving several HIV patients with a history of average ARV therapy duration of more than 17 years without treatment failure, integrase inhibitor and viral suppression, no integrase-resistant mutation was found except for mutation 74I in a patient who received an EGV regimen. Therefore, further randomized studies are required to assess the efficacy of DTG as a standalone ARV therapy, especially in INSTI-naive patients [51].

In the VIKING-4 clinical trial study, heavy treatment-experienced patients with treatment failure against RAL regimen were administered DTG functional monotherapy with placebo for seven days followed by DTG + optimized background regimen for 48 weeks. It was concluded that DTG functional monotherapy could contribute to the development of DTG resistance and background drugs and also to virological failure. The majority of patients who underwent primary INSTI substitution at baseline have T66A/I/K, Y143 R/C/H/G, Q148 H/R, and N155H mutation. This necessitates the need for further studies with shortened duration of DTG monotherapy to minimize the risk of developing resistance [52].

INSTI DTG as triple therapy dolutegravir/lamivudine/abacavir (DTG/3TC/ABC) in experienced-treatment patients with a pre-existing M184V/I mutation is at risk for virological failure due to the development of resistance to all types of INSTI [14]. The M184V/I mutation is an NRTI DRM that can reduce susceptibility to regimens containing 3 TC or FTC up to 100 folds, as well as reduce the susceptibility to ABC. Despite a study finding that patients with pre-existing M184V/I mutation who received triple therapy DTG/3TC/ABC did not exhibit risk of virological failure, viral load monitoring was still needed in patients who received the triple therapy regimens [14,53].

## 7. Conclusion

Primary or major integrase mutations against INSTI ARV are still rare, but minor (secondary) or polymorphic mutations that can affect INSTI resistance are often found in treatment-naive, INSTI-naive, and INSTI-experienced treatment patients. The presence of secondary mutations, if found together with other mutations, can increase the level of resistance to INSTI. In various *in vitro* and *in vivo* studies, several integrase mutations exhibit different genetic barriers to INSTI resistance between various non-B subtypes and subtype B of HIV-1. Hence, in specific non-B subtypes, the presence of integrase mutation can have different effects on INSTI resistance compared with other subtypes. Therefore, due to the high variability of HIV-1, ongoing surveillance is necessary to assess the effects of each integrase mutation on the various subtypes of HIV-1.

Considering the fact that DTG belongs to the INSTI group with the highest genetic barrier and the recommendation of its use as first and second-line regimens for both naive and experienced-treatment patients, further clinical trials are needed to determine the risk of drug resistance following the administration of DTG monotherapy. In experienced-treatment patients who received a triple therapy regimen containing DTG, it is necessary to identify the presence of NRTI DRM due to the risk of developing resistance to INSTI.
